# Cadmium Stress Disrupts the Endomembrane Organelles and Endocytosis during *Picea wilsonii* Pollen Germination and Tube Growth

**DOI:** 10.1371/journal.pone.0094721

**Published:** 2014-04-10

**Authors:** Xiaoxia Wang, Yuan Gao, Yu Feng, Xue Li, Qian Wei, Xianyong Sheng

**Affiliations:** College of Life Sciences, Capital Normal University, Beijing, China; Iowa State University, United States of America

## Abstract

As one of the most severe pollutants, cadmium has been reported to be harmful to plant cells, but the effects of cadmium on gymnosperm pollen germination and tube growth and the mechanism of this involvement are still unclear. Here, we report that cadmium not only strongly inhibited *P. wilsonii* pollen germination and tube growth, but also significantly altered tube morphology in a dose-dependent manner. Time-lapse images obtained with a laser scanning confocal microscope revealed that endocytosis was dramatically inhibited by cadmium stress. Further investigation with ER-Tracker dye indicated that cadmium stress reduced the number of the Golgi apparatus, and induced dilation of ER. Additionally, Lyso-Tracker staining showed that cadmium distinctly promoted the formation of acidic organelles in pollen tubes, likely derived from the dilated ER. Taken together, our studies indicated that *P. wilsonii* pollens were highly susceptible to cadmium stress, and that cadmium stress strongly inhibited pollen germination and tube growth by disrupting the endomembrane organelles, inhibiting endo/exocytosis, and forming acidic vacuoles, resulting in swollen tube tips and irregularly broadened tube diameters. These findings provide a new insight into the effects of cadmium toxicity on the tip growth of pollen tubes.

## Introduction

Rapid industrialization and urbanization in developing countries has resulted in a significant increase in environmental pollution. For example, most parts of northern and eastern China are frequently enveloped by heavily polluted haze during the spring and winter [1], [2], in which cadmium is one of the severe metal contaminants. Although cadmium is a nonessential element, and no specific uptake systems has been reported, it can be easily taken up by plants via transporters/channels for essential cations [3]–[5]. For example, it was reported that cadmium can competitively permeate into guard cells through calcium channels [6]. Besides, ZIP family of metal transporters, the main iron uptake system in *Arabidopsis thaliana* root, is responsible for cadmium influx into root cells [7]. What's more, once been adsorbed and accumulated in the plant tissues, cadmium can not only severely decrease the productivity and quality of crops, but also readily enter the food chain, and ultimately endanger the health of both humans and animals [4], [8]–[10]. Hence, increasing attention is being paid to understand the effects of cadmium pollution on plants.

Pollen grains are male reproductive structures whose function is to transport and discharge sperms into the embryo sac [11]–[13]. To achieve this, pollen grains of cross-pollination species, especially anemophilous plants such as gymnosperms, have to travel through the air from one tree to another [13]. During this period, they are exposed to heavy metal pollutants, including cadmium that float in the air as dusts, fumes, mists, and vapors [13]–[16]. Previous reports had indicated that cadmium strongly inhibited pollen germination and tube growth [17]–[20]. But nearly all of the data available at present are focused on angiosperm pollen grains; and little attention has been paid to the possible effects of cadmium on gymnosperm pollen germination and tube growth. Furthermore, germination of pollen grains is a complex biological event, during which a number of factors and activities are required to be integrated in space and time [11]–[13], [21]. However, current information appears to be insufficient in providing complete knowledge of the mechanism by which cadmium inhibits pollen germination and tube growth. Importantly, no attention has been paid to the possible effects of cadmium on the endomembrane system and endo/exocytosis, which are closely linked to the tip growth of pollen tubes [11], [21].

To extend our knowledge on the effects of cadmium on gymnosperm pollen tube growth, we examined the germination rate, tube length and morphology of *Picea wilsonii* pollen grains treated with various concentrations of cadmium nitrate (Cd(NO_3_)_2_). Additionally, we present data on the cadmium-induced alterations in the endomembrane organelles and endocytosis, thereby providing further insights into the mechanism by which cadmium affects the growth of pollen tubes.

## Materials and Methods

### Ethics statement

Mature *P. wilsonii* pollen grains were collected from trees growing in the botanical garden of the Institute of Botany, Chinese Academy of Sciences (N 39.987342°,E 116.210691°), and stored at −20°C until use. This study did not involve any endangered or protected species, and no specific permissions were required.

### Pollen culture

*In vitro* pollen culture was performed according to our previous reports [11], [13]. Briefly, pollen grains (1 mg mL^−1^) were suspended in the standard germination medium containing 12.5% (w/v) sucrose, 0.01% (w/v) H_3_BO_3_, and 0.01% (w/v) CaCl_2_ and cultured on a shaker (121 rpm) at 24°C. Cadmium treatment was performed by adding 10, 20, or 40 µM Cd(NO_3_)_2_ into the standard germination medium from the initiation of incubation.

### Determination of pollen germination and tube length

Germination rate and tube length were determined by scoring at least 300 randomly chosen pollen grains of each sample using a ZEISS Axiovert 200 M microscope equipped with a Q imaging RETIGA-SRV CCD. Pollen grains were considered as germinated when the tube length was greater than the diameter of the pollen grain [11]–[13]. Tube length and width were measured using Image-Pro Plus 7.0 (MediaCybernetics, Bethesda, MD, USA).

### FM4-64 staining

To better understand the possible effects of cadmium on endocytosis, both control and 10 µM cadmium-treated samples were stained with 2.5 µg mL^−1^ FM4-64 (Invitrogen, final concentration) [21], and time-lapse images were obtained using a Zeiss 5 Live laser scanning confocal microscope, with an excitation wavelength of 561 nm and emitted wavelength of LP 575 nm. Laser power and channel settings were kept identical for all samples to make the results comparable.

### ER-Tracker and Lyso-Tracker staining

To better understand the possible effects of cadmium on endomembrane organelles, both control and 10 µM cadmium-treated samples were stained with 50 µM ER-Tracker Green (Invitrogen, final concentration) for 10 mins, or with 10 µM Lyso-Tracker Green (Invitrogen, final concentration) for 30 mins, respectively [22]. All samples were examined using a Zeiss 5 Live laser scanning confocal microscope, with an excitation wavelength of 488 nm and emitted wavelength of BP 494–555 nm. The obtained images were subsequently analyzed using Zen 2009 (Zeiss, Germany).

### Statistical analysis

All experiments were performed at least in triplicate. Oneway ANOVA was used to compare the difference between the control and cadmium-treated pollen tubes. P≤0.05 was considered were taken as statistically significant.

## Results

### Cadmium reduced pollen germination and prevented tube elongation

**Figure 1:**
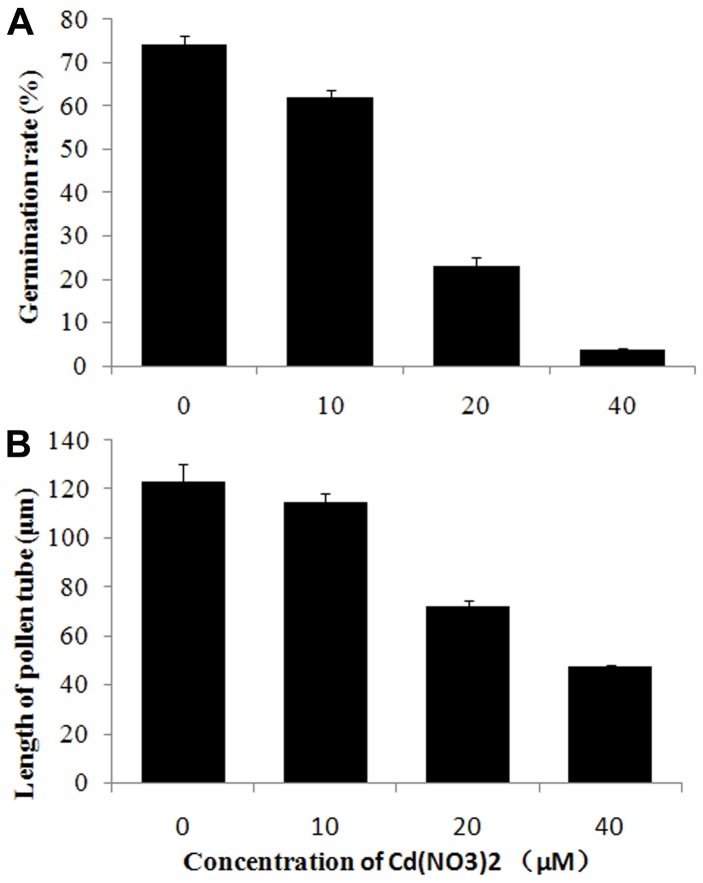
Effects of cadmium on pollen germination rate and tube length. Germination rate (A) and tube length (B) of pollen grains/tubes treated with 0, 10, 20, or 40 µM Cd(NO_3_)_2_ for 20 h, respectively. All data represent mean ± SD of three independent experiments.

*In vitro* germination of *P. wilsonii* pollen under control conditions is characterized by a long lag phase (about 12 h), after which the tube slowly emerges and elongates at an average rate of about 10 µm h^−1^ [11]–[13]. Microscopic evaluation revealed that nearly 75% of all 20 h-cultured pollen grains had germinated, with an average tube length of about 123.08 µm (Fig. 1), indicating a superior vigor of the pollen grains used in the present study.

In contrast, cadmium treatment significantly inhibited *P. wilsonii* pollen germination and tube growth in a dose-dependent manner (Fig. 1). In fact, microscopic evaluation revealed that when treated with 10 µM cadmium for 20 h, only 61.9% of pollen grains had germinated with an average tube length of 114.83 µm (p<0.01). With increasing cadmium concentration, the pollen germination rate was drastically reduced to 23% (for 20 µM cadmium) and 3.7% (for 40 µM cadmium) (p<0.001). Additionally, the average tube lengths were similarly reduced to 72.3 µm (for 20 µM cadmium) and 47.6 µm (for 40 µM cadmium) (p<0.001).

### Cadmium induced morphological changes in pollen tubes

**Figure 2:**
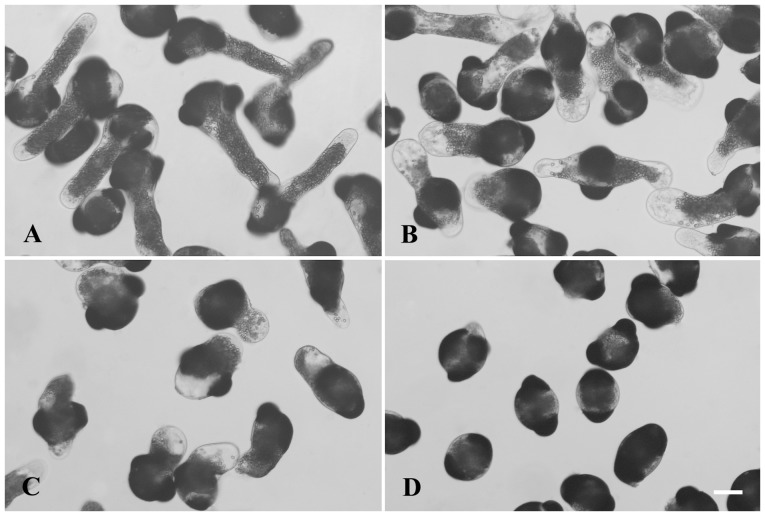
Effects of cadmium on the tube morphology. Pollen tubes were treated with 0 (A), 10 (B), 20 (C), or 40 (D) µM Cd(NO_3_)_2_ for 20 h, respectively. Images show that cadmium not only inhibited pollen germination and tube growth, but also induced morphological alterations, including cytoplasmic vacuolization, swollen tips and irregular tube diameters. Bars  = 50 µm.

Normal *P. wilsonii* pollen tubes were typically characterized by a uniform diameter, with amyloplasts distributed throughout the tube except at the apex (Fig. 2A). Cadmium stress strongly affects the typical morphological organization of pollen tubes, particularly in the apical and subapical regions. The most obvious phenomena were swollen tips and irregularly broadened tube diameters (Fig. 2B–C). Microscopic evaluation revealed that the average width of 10 µM cadmium-treated tubes was about 62 µm (n = 139 pollen tubes), whereas the data of control tubes was about 39 µm (p<0.001). Besides, cytoplasmic vacuolization occurring in the subapical regions and/or shoots of tubes was frequently observed in the cadmium-treated pollen tubes (Fig. 2B–C), even in the short tubes that had barely emerged from the 40 µM cadmium-treated pollen grains (Fig. 2D).

### Cadmium stress inhibited endocytosis in pollen tubes

**Figure 3:**
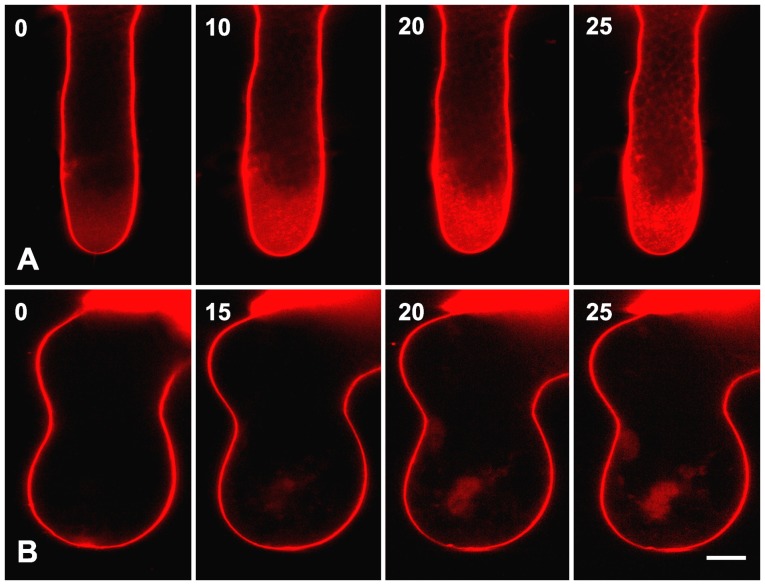
Effects of cadmium on the endocytosis. Control (A) and 10 µM cadmium-treated (B) pollen tubes were labeled with FM4-64, and time-lapse images were obtained using a Zeiss 5 live confocal microscope. In total, 9 control tubes and 15 cadmium-treated tubes were examined. No obvious individual differences were observed within control or cadmium-treated tubes, respectively. Images show that cadmium strongly inhibited endocytosis occuring at the tip of pollen tubes. Bars  = 10 µm.

Since the rate of pollen tube growth depends on efficient cytosis to provide enough new plasma membrane and cell wall components [23]–[25], we therefore speculated that cadmium could disrupt endocytosis and/or exocytosis during *P. wilsonii* pollen tube elongation. To examine this possibility, FM4-64, a fluorescent dye widely used in studying endocytosis and exocytosis [21], [25], was used in the present study. Time-lapse images revealed that the internalization of FM4-64 into *P. wilsonii* pollen tubes followed a strict time sequence (Fig. 3A). At first, only plasma membrane showed bright FM4-64 fluorescence immediately after adding the dye into the germination medium. With increasing time of incubation, fluorescent signals were gradually detectable in the clear zone of pollen tubes, indicating the occurrence of endocytosis in this region. Usually, the typical staining pattern of FM4-64 in the entire clear zone of control tubes can be observed within 30 mins (Fig. 3A). In contrast, although FM4-64 fluorescence could also be observed at the plasma membrane of 10 µM cadmium-treated pollen tubes, the dye was internalized more slowly into cadmium-treated tubes as compared to the control tubes (Fig. 3B), suggesting that endocytosis was disrupted in pollen tubes in response to cadmium stress. Consequently, although the cadmium-treated tubes were stained with FM4-64 for the same time as the control tubes, only a few fluorescent signals were detected in the cytoplasm, usually aggregated in the subapical regions (Fig. 3).

### Cadmium stress disrupted endomembrane organelles in pollen tubes

The contents of exocytotic vesicles are usually initiated in the endoplasmic reticulum (ER), where they are assembled and travel through the Golgi apparatus towards the plasma membrane or vacuoles [11], [26]. To verify the possibility that cadmium stress could alter the distribution and morphology of the endomembrane organelles, ER-Tracker was used in our subsequent experiments.

**Figure 4:**
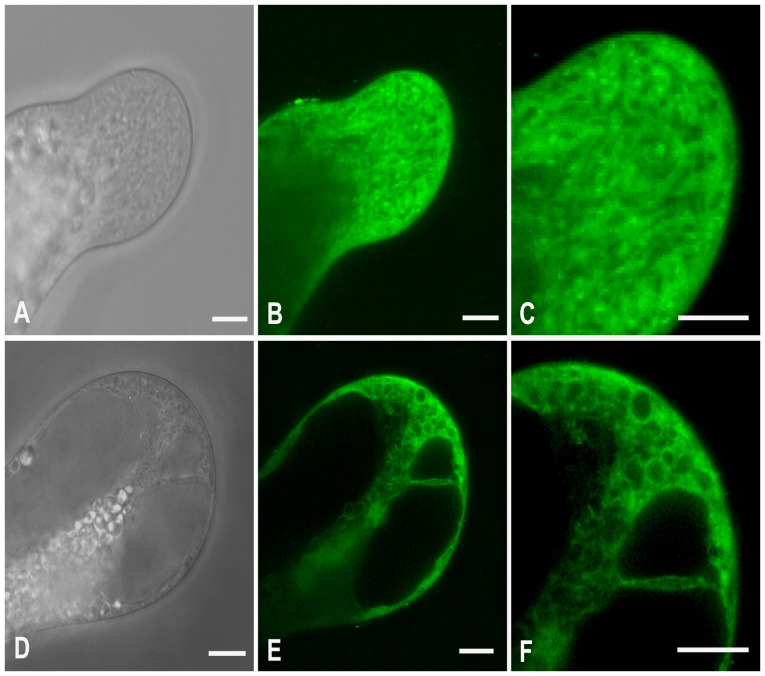
Effects of cadmium on the ER and Golgi apparatus. Control (A–C) and 10 µM cadmium-treated (D–F) pollen tubes were labeled with ER-Tracker Green. Differential interference contrast images (A, D) and corresponding fluorescent images (B, E) were obtained using a Zeiss 5 live confocal microscope. In total, 11 control tubes and 20 cadmium-treated tubes were examined. No obvious individual differences were observed within control or cadmium-treated tubes, respectively. Images C and F represents magnified views of the tip regions in images B and E, respectively. Images show that cadmium reduced the amount of ER and number of Golgi apparatus, and induced vacuole-like organelles. Bars  = 10 µm.

Confocal observation indicated that the entire clear zone of control tubes was filled with the ER-Tracker dye that existed mainly in a reticulate network and occasionally in some spherical shape structures with diameters ranging from 0.3 to 1.5 µm (Fig. 4A–C). Both the reticulate network and spherical shape structures showed active motility, mainly in a fountain pattern (Movie S1). Based on our previous TEM observations on *P. wilsonii* pollen tubes [11], and ER-Tracker staining in living hyphae of *Pisolithus tinctorius* [27], the reticulate network in pollen tubes was undoubtedly ER, and the small spherical shape structures were presumably the Golgi apparatus. In contrast, 10 µM cadmium significantly reduced the amount of reticulate network (Fig. 4D–F). Besides, the spherical shape structures frequently observed in control tubes seem miss in treated tubes. Instead, many ring-like organelles, with various diameters ranging from 1.3 to 4.5 µm, were usually labeled with bright fluorescence (Fig. 4D–F). These data were partially reminiscent of previous studies, which reported that ER is a major target of cadmium toxicity [28], [29].

**Figure 5:**
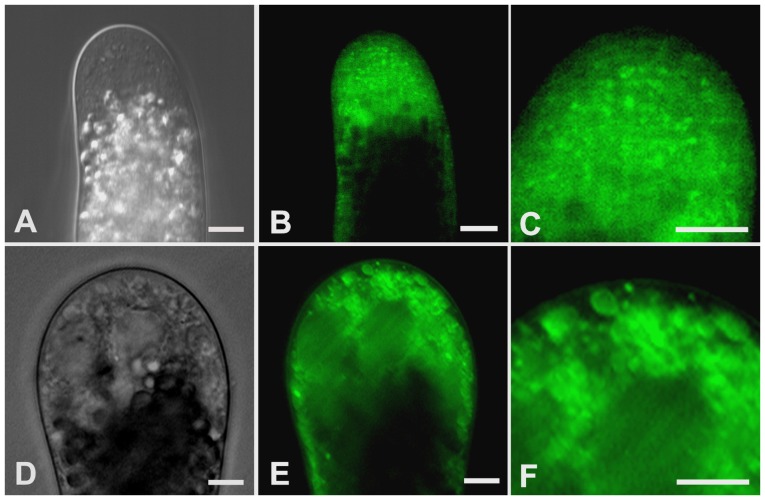
Effects of cadmium on the formation of acid vacuoles. Control (A–C) and 10 µM cadmium-treated (D–F) pollen tubes were labeled with Lyso-Tracker Green. Differential interference contrast images (A, D) and corresponding fluorescent images (B, E) were obtained using a Zeiss 5 live confocal microscope. In total, 12 control tubes and 10 cadmium-treated tubes were examined. No obvious individual differences were observed within control or cadmium-treated tubes, respectively. Images C and F represent magnified views of the tip regions in images B and E, respectively. Images show that cadmium promoted the formation of acidic organelles in pollen tubes. Bars  = 10 µm.

To further elucidate the cadmium-induced cytoplasmic vacuolization in pollen tubes, Lyso-Tracker Green, a dye that selectively accumulates in cellular compartments with low internal pH, was utilized in the subsequent experiment. The results showed that Lyso-Tracker in control tubes was mainly found in some small isolated punctate structures within one micron in diameter, which might indicate the existence of some small vacuoles with acid hydrolase. They were randomly distributed in the whole clean zone, except for the apical region (Fig. 5A–C). In cadmium treated tubes, numerous vacuole-like organelles with various diameters, ranging from 1.2 to 4.2 µm, were labeled with brighter fluorescence. Those Lyso-Tracker-labeled organelles were distributed throughout the entire tip of the cadmium-treated tubes, even in the extreme apex (Fig. 5D–F).

Given that Lyso-Tracker could also label autophagy [22], part of the cadmium-treated samples were stained with 125 µM Dansylcadaverine (MDC, Sigma). Results showed that no fluorescence was observed in the MDC-stained tubes (data not shown), indicating that cadmium treatment did not induce autophagy.

## Discussion

To achieve their functions of pollination and fertilization, pollen grains of cross-pollination species have to be exposed to air. During this period, they are prone to accumulate heavy metal pollutants, including cadmium and lead, that exist in the atmosphere [4], [13]. Cadmium can competitively enter cells [5], and have highly toxic effects on both plants and animals [3], [8]. Especially, it was reported that cadmium strongly inhibited angiosperm pollen germination and tube growth [17]–[20], [30], [31]. In the present study, we found that cadmium not only strongly prevented *P. wilsonii* pollen germination and tube growth in a dose-dependent manner (Fig. 1), but also caused significant morphological alterations, including cytoplasmic vacuolization, swollen tips and irregular tube diameters (Fig. 2). Our data is consistent with previous reports [17]–[20], [30], [31], confirming that pollen tubes are extremely sensitive to cadmium stress.

Cadmium was believed to have the highest pollution index, and was chosen as the top priority monitoring goal in China [9], [32]. It was reported that atmospheric deposition of cadmium in farmland soils ranged from 0.04 to 2.5 mg/m^2^/yr [9]. The contaminants suspended in the atmosphere are likely to come into contact with plant surfaces. In fact, up to 1.63 mg kg^−1^ cadmium was detected in the surfaces of leaves of rose plants growing near the 4^th^ ring road in Beijing [33]. By accumulation in the pollen grains and/or the stigmas, cadmium can reach the concentration which can directly inhibit the germination and growth of pollen tubes [19]. Thus, our data are consistent with and confirm previous reports that pollen grains can be used as a sensitive system for biomonitoring of heavy metals in urban environment [16], [19]. Besides, we found that hardly any *P. wilsonii* pollen grains had germinated when treated with 40 µM cadmium. This concentration is much lower than that reported for *L. longiflorum* and *N. tabacum* [19], but is slightly higher than that reported for *P. sylvestris* [15]. This difference in sensitivity to cadmium could be explained by the prolonged exposure of *P. wilsonii* pollens to cadmium, since they need a long lag phase, and grow far more slowly than angiosperm pollen tubes [11], [13]. Since gymnosperm pollen grains are more sensitive to cadmium stress as compared to angiosperms, we speculate that gymnosperm pollen grains, such as *P. wilsonii*, might be more competent plant indicators of air pollution.

It has long been appreciated that cadmium strongly inhibits pollen germination and tube growth [17]–[20], [30], [31]. Yet, the precise mechanism underlying this phenomenon still remains insufficient. In the present study, our data revealed that cadmium induced ring-like ER and reduced the number of Golgi apparatus (Fig. 4), indicating that endomembrane organelles, especially ER, might be a major target of cadmium toxicity in *P. wilsonii* pollen tubes, which was indeed observed in other cells. For example, it was reported that cadmium-induced ER stress and toxicity in yeast were direct consequences of cadmium accumulation in the ER [28]. More recent evidence indicated that cadmium induced dilation of ER, and subsequent cytoplasmic vacuolization in BY-2 cells [34]. Given that the ER is an entrance compartment to the secretory pathway, and that exocytotic vesicles are derived directly from the Golgi apparatus [26], the disorders in the endomembrane organelles meight be the most probable explanation for cadmium induced inhibition on pollen tube growth.

Besides, tip growth of the pollen tube is a typical polarized growth, resulting from continued fusion of secretory vesicles with the plasma membrane at the apex of the pollen tube [23], [24]. The amount of membrane delivered by exocytosis is in excess of that required for the pollen tube growth, therefore the balance between exocytosis and endocytosis is essential for normal pollen tube growth [21], [25]. In the present study, time-lape images revealed that the uptake of FM4-64 dye occurred much slower in cadmium-treated tubes (Fig. 3), indicating that cadmium inhibited endo/exocytosis in pollen tubes. Previous reports indicated that the dynamic calcium gradient in pollen tubes plays a key role in the endo/exocytosis in pollen tubes [11]. While cadmium can competitively enter plant cells by calcium transporters/channels such as calmodulin [5]. Therefore, cadmium induced abnormal calcium gradient might be another possibility that still cannot be excluded. Anyhow, our data are consistent with our theoretical prediction, and confirm that cadmium stress strongly disrupted the secretory pathway, which would inevitably inhibit pollen germination and tube growth, as observed in our study.

Plant vacuoles are very important organelles. Apart from maintaining appropriate turgor and proper pH level, they also function in storage of metabolic products and digestion of cytoplasmic constituents [35]. A previous research has indicated that cadmium induced significant cytoplasmic vacuolization, even at the apex of pollen tubes [19]. Accumulation of cadmium in the vacuoles was indeed detected in the root cells of *Allium sativum* using electron energy loss spectroscopy [36]. Cadmium-induced cytoplasmic vacuolization in BY-2 cells was also reported [34]. In the present study, cadmium-induced cytoplasmic vacuolization was observed in *P. wilsonii* pollen tubes (Fig. 2), indicating that plant vacuoles are important organelles involved in cellular responses to cadmium stress [35]. Given that plant cell growth is driven by internal turgor pressure, and is restricted by the ability of the cell wall to extend under these forces [11], cadmium-induced cytoplasmic vacuolization might be one of the most important factors in the formation of swollen tube tips and irregularly broadened tube diameters.

In summary, our data revealed that *P. wilsonii* pollens were highly susceptible to cadmium stress. Cadmium strongly disrupted endomembrane organelles, and induced cytoplasmic vacuolization in pollen tubes. Consequently, reduction of endo/exocytosis and formation of acidic vacuoles led to the disruption of tip growth, providing a new insight into the mechanism by which plant cells respond to cadmium stress.

## Supporting Information

Movie S1 **Time-lapse images (about one frame per sec) of a control tube stained with ER-Tracker were captured using a ZEISS 5 live confocal microscope.** The obtained images were converted to an avi file (about 20 frames per sec) using Zen 2009 (Zeiss, Germany).
(AVI)
